# Quantitative and organisational changes in mature extracellular matrix revealed through high-content imaging of total protein fluorescently stained *in situ*

**DOI:** 10.1038/s41598-017-10298-x

**Published:** 2017-08-30

**Authors:** Gill Holdsworth, Hélène Bon, Marianne Bergin, Omar Qureshi, Ross Paveley, John Atkinson, Linghong Huang, Roohi Tewari, Breda Twomey, Timothy Johnson

**Affiliations:** UCB Pharma, Slough, UK

## Abstract

Fibrosis is a common driver of end-stage organ failure in most organs. It is characterised by excessive accumulation of extracellular matrix (ECM) proteins. Therapeutic options are limited and novel treatments are urgently required, however current cell-based high-throughput screening (HTS) models to identify molecules affecting ECM accumulation are limited in their relevance or throughput. We report a novel sensitive approach which combines *in situ* fluorescent staining of accumulated decellularised ECM proteins with automated high-content microscopy. Using this method to measure ECM accumulation in a kidney cell model, we demonstrated good agreement with established radiolabelled amino acid incorporation assays: TGFβ1 delivered a potent pro-fibrotic stimulus, which was reduced by TGFβ antibody or the anti-fibrotic nintedanib. Importantly, our method also provides information about matrix organisation: the extent of ECM accumulation was unaffected by the BMP antagonist Gremlin-1 but a pronounced effect on matrix fibrillar organisation was revealed. This rapid, straightforward endpoint provides quantitative data on ECM accumulation and offers a convenient cross-species readout that does not require antibodies. Our method facilitates discovery of novel pro- and anti-fibrotic agents in 384-well plate format and may be widely applied to *in vitro* cell-based models in which matrix protein deposition reflects the underlying biology or pathology.

## Introduction

The extracellular matrix (ECM) is a complex mix of proteins, proteoglycans and fluid, which was traditionally considered as a relatively inert material that provided a structural framework for cell binding, but is now recognised as an important regulator of cellular and tissue function^1,2^. Its mechanical properties vary across different tissues and organs, and the stiffness and elasticity reflect differences in its constituent components, allowing the matrix to serve many specialised roles. The ECM acts as site of growth factor storage and activation, and forms direct interactions with cell-surface molecules such as integrins, thereby delivering extracellular and environmental feedback to the surrounding cells. It actively influences the biology of the cells in its local environment, regulating cellular contraction, migration, proliferation, differentiation and apoptosis.

Mature ECM is a highly dynamic structure which is composed of a large number of proteins that are maintained in a homeostatic balance by controlled continuous remodelling, with the synthesis and deposition of ECM components on one side and the activity of clearance enzymes such as matrix metalloproteinases on the other^2^. Aberrant ECM remodelling after tissue injury leads to a fibrotic response in which the rate of matrix protein synthesis and deposition outweighs clearance, and the composition of the ECM is altered through increased collagen expression, leading to ECM accumulation and scarring of the parenchyma. The resultant decline in the elasticity and function of the affected organ may ultimately lead to its failure; consequently, fibrosis presents a significant burden of morbidity and mortality^1,3^.

Methods for monitoring changes in the ECM *in vitro* typically involve the measurement of transcripts encoding immature protein precursors of key ECM components, such as collagens and fibronectin, or detecting changes in markers of epithelial to mesenchymal transition (EMT) at the protein level such as smooth muscle actin, vimentin or β-catenin. However, surrogate mRNA readouts do not fully capture the homeostatic balance of deposition and degradation which ultimately determines the total matrix accumulation, while EMT may not in itself dramatically affect the ECM turnover that directly leads to scar tissue. The complex nature of ECM regulation means it is important to monitor the accumulation of the mature matrix if the underlying biology is to be uncovered, or if the mechanism of action of novel anti-fibrotic agents is to be understood. Therefore, effective and biologically relevant approaches which are able to detect pathological changes in the balance of ECM are required.

Methods to assess accumulation of mature ECM in *in vitro* cell-based models include those which quantitate the collagen content of the deposited matrix, such as Sirius red staining or hydroxyproline analysis^4^. The gold-standard method to measure ECM accumulation is via quantitation of radioactivity incorporated into the matrix of cells grown in the presence of radiolabelled amino acids. This approach is sensitive and quantitative; however it makes assumptions that the incorporation of the labelled amino acids is unbiased and uniform. Standard radioactive ECM deposition protocols are expensive to perform and can only be undertaken in appropriately equipped laboratories. The method typically utilises large surface areas and hence tends to be very low-throughput and although we have successfully miniaturised this assay, the approach still presents challenges associated with using radioactive solutions in robotic liquid handling systems. Furthermore, the radioactive ECM endpoint does not yield any information regarding the organisation of the mature matrix.

More recently, sensitive immunofluorescence methods have been developed by ourselves and others, which use antibodies to label specific matrix proteins, such as type I collagen, or fibronectin, in fixed decellularised ECM^5,6^. This approach permits high-content analysis of ECM, however there are significant limitations to how easily this method may be applied. In addition to the requirement for specific antibodies which cross-react with the protein(s) of interest, and which recognise the conformation of that protein *in situ* in the ECM, the number of ECM proteins which can be monitored is also restricted by the multiplex capability of the high-content microscope being used.

Using an *in vitro* model of renal epithelial cells, we exploited the high sensitivity of a fluorescent protein dye that is typically used to stain 2D proteomics gels to label mature deposited decellularised ECM *in situ*, thus developing a novel high-content imaging method. In order to provide a robust system for the evaluation of this novel endpoint, we elected to use TGFβ as a potent pro-fibrotic stimulus, and demonstrated that the signal could be positively or negatively modulated using well known inducers and inhibitors of matrix deposition. This approach showed good agreement with the traditional method of measuring radiolabelled amino acid incorporation in deposited ECM, and importantly, also revealed changes in the fibrillar organisation of the mature ECM. Fluorescent staining of the total deposited ECM *in situ* offers an alternate approach which is easy to perform and low cost, and since the technique is not antibody-based, it can be universally applied to the measurement of total matrix deposited by cells from any species.

## Results

### TGFβ1 stimulates production of ECM components

**Figure 1:**
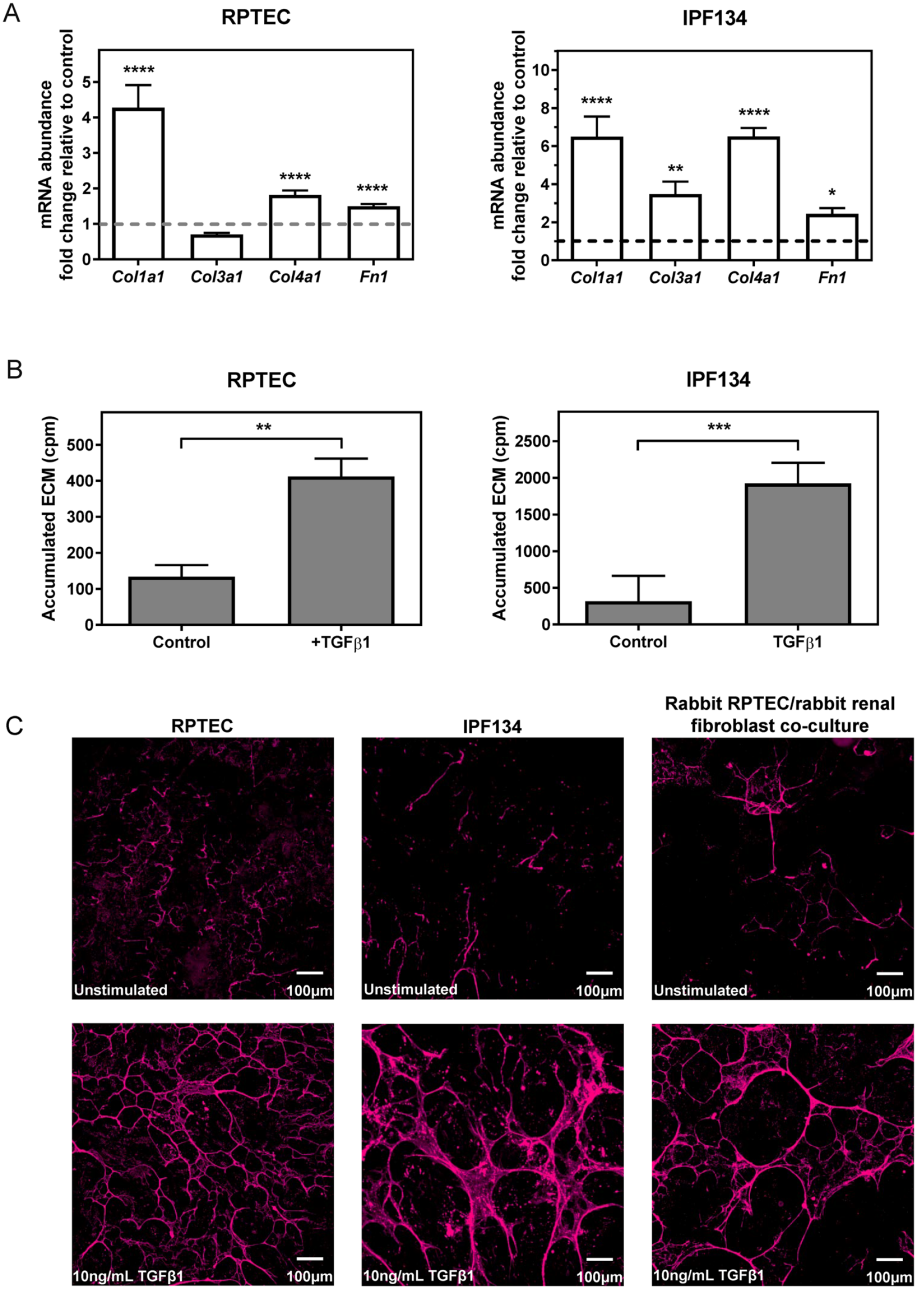
TGFβ1 stimulates the expression of ECM components in human and rabbit primary cells. (**A**) Selected ECM mRNA transcripts were measured using qRT-PCR in human RPTEC or human IPF134 following 48 hr stimulation with TGFβ1 (10 ng/mL). Data show transcript abundance relative to the unstimulated control (indicated by the dashed line) and are shown as mean ± SD of four (RPTEC) or seven (IPF134) independent experiments. ****P < 0.0001; ****P < 0.001; **P < 0.01; Student’s t-test. (**B**) The accumulation of ECM in human RPTEC (i) or human IPF (ii) cells stimulated with 10ng/mL TGFβ1 was evaluated via measurement of the incorporation of ^14^C-labelled amino acids into the deposited ECM. Data presented are the mean ± SD of three independent experiments. **P < 0.01; Student’s t-test. (**C**) Increased mature ECM accumulation following treatment of cells with a pro-fibrotic stimulus can be visualised following *in-situ* fluorescent staining using the Flamingo dye. Cells from three different systems (primary human RPTEC; primary human IPF134; co-culture of primary rabbit RPTEC with primary rabbit renal fibroblasts) were cultured in the absence (top) or presence (bottom) of 10 ng/ml TGFβ1 before decellularised matrix was fixed and stained *in situ* with Flamingo fluorescent dye. Images show a single field.

TGFβ is recognised as one of the major drivers of organ fibrosis^7–9^. Initially, we demonstrated the ability of TGFβ1 to deliver a potent fibrotic stimulus *in vitro* using two typical endpoints with different human primary cell types from two organs: renal proximal tubular epithelial cells (RPTEC) and fibroblasts originating from idiopathic pulmonary fibrosis lung (IPF134). Expression of key ECM genes was determined using qRT-PCR (Fig. 1A). RPTEC showed strong (1.5–4.3 fold) up-regulation of transcripts encoding *collagen 1a1*, *collagen 4a1* and *fibronectin* following stimulation with TGFβ1 for 48 hr, whilst *collagen 3a1* was slightly decreased. IPF134 fibroblasts typically showed larger changes in response to TGFβ1 and the abundance of *collagen 1a1, collagen 3a1*, *collagen 4a1* and *fibronectin* was increased 2.4–6.5 fold.

The traditional methods to measure accumulation of mature ECM use analytical quantitation of hydroxyproline or incorporation of radiolabelled amino acids in the matrix^4^. Both methods require large surface areas and are typically carried out in 20 cm dishes. We successfully miniaturised the radioactive endpoint so that it was amenable to screening (summarised in Supplementary Fig. 2). Measurement of total extracellular matrix via incorporation of ^14^C radioactive amino acids (Fig. 1B) demonstrated the effect of TGFβ1 on ECM deposition by RPTEC and fibroblasts. RPTEC deposited small amounts of mature ECM under basal conditions and treatment with TGFβ1 promoted a considerable (>3 fold) increase in ECM deposition. IPF134 cells exhibited greater levels of basal ECM deposition compared with RPTEC, and this was further increased (approximately 6.5 fold) following stimulation with TGFβ1.

Together, these data demonstrate the ability of TGFβ1 to deliver a potent fibrotic stimulus to different cell types from two different organs, which is reflected both at the level of mRNA abundance of key matrix components, and accumulation of mature matrix as determined by the measurement of incorporation of radioactive amino acids into the matrix.

### Visualisation of deposited ECM using a fluorescent total protein stain

Whilst the deposition of mature ECM can be monitored through the incorporation of radioactive amino acids, this methodology does not permit visualisation of the matrix *in situ*. It is possible to perform immunofluorescence on proteins contained in the deposited decellularised matrix^5,6^ and we wanted to know whether a fluorescent total protein stain could be used to visualise the proteinaceous components of deposited ECM. Similar to the radioactive endpoint, this would permit an unbiased determination of the matrix without the need to use radiolabelled amino acids, but like the immunofluorescence approaches would also provide visual information about the nature of the ECM.

The small amounts of ECM laid down by cells per unit area, which we estimated to be in the region of low ng total protein per well of a 96-well plate, mean that such a stain would need to be highly sensitive and should ideally have a large linear response. Reagents used to visualise small amounts of protein on 2D proteomics gels are available so we wondered whether any of these gel stains could be applied to staining deposited ECM *in situ*. Some proteomics gel stains (e.g. Deep Purple) are dependent on SDS denaturation of the proteins being separated so are not suitable for our intended purpose of staining matrix proteins *in situ*. Other fluorescent protein stains are used to stain proteins separated via native PAGE, and also met our criteria of high sensitivity and wide dynamic response. We tested the ability of two such fluorescent gel stains: SYPRO Ruby and Flamingo, to stain mature deposited ECM *in situ*. RPTEC were cultured in the presence or absence of TGFβ1 for 7 days, after which cells were removed using ammonium hydroxide. The accumulated decellularised ECM was fixed using 50% methanol supplemented with 7.5% acetic acid before being stained with each dye. Fluorescence microscopy revealed the presence of a fibrillar matrix which was detected following staining with each of the reagents, however SYPRO Ruby exhibited pronounced photobleaching (Supplementary Fig. 3) and so was not used in further experiments. Figure 1C shows the increase in mature ECM accumulation following stimulation of RPTEC or IPF134 cells with TGFβ1, detected by *in situ* fluorescent staining of ECM using the Flamingo fluorescent stain. *In situ* fluorescent staining of deposited decellularised ECM yielded images which were highly comparable to those obtained by imaging-based immunofluorescence of the key ECM proteins collagen type I/III and fibronectin (Supplementary Fig. 4), indicating that it is proteinaceous ECM which is labelled by the Flamingo stain.

Importantly, we also demonstrated that this approach is not limited to detection of matrix laid down by human cells. Figure 1C shows ECM deposited by a co-culture of primary rabbit RPTEC with primary rabbit kidney fibroblasts in response to TGFβ1 stimulation and stained *in situ* with the Flamingo fluorescent stain. Together, these data demonstrate the applicability of this *in situ* staining approach to detect matrix deposited by cells originating from different organs and across species.

### Fluorescent staining of ECM *in situ* is comparable to incorporation of radiolabelled amino acids as a method to determine the extent of ECM deposition

**Figure 2:**
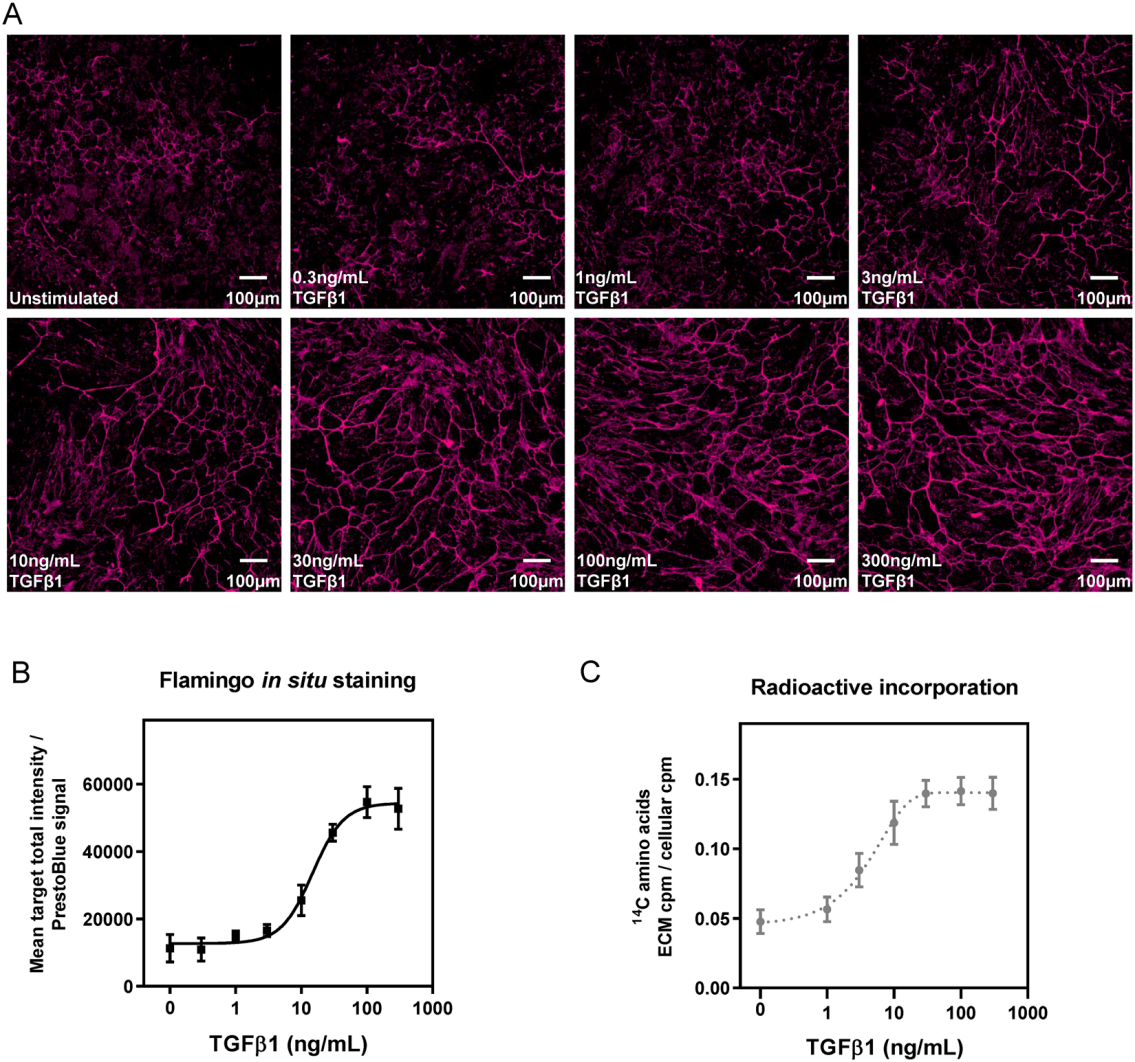
*In situ* fluorescent staining can be used to measure dose-dependent effects of TGFβ1 on mature ECM accumulation. (**A**) Human RPTEC were stimulated with different concentrations of TGFβ1. The decellularised matrix was fixed and stained *in situ* with Flamingo fluorescent dye. Images are representative of a single field. (**B**) High-content analysis of the data collected in A. was performed using the Cellomics “Cell Health Profiling” bioapplication to obtain fluorescence intensity data for decellularised accumulated mature ECM stained *in situ* with Flamingo fluorescent dye. The PrestoBlue signal was used to normalise for differences in cell number. The dose-response curve shows mean target fluorescence intensity normalised to the PrestoBlue signal (mean ± SD of 6 replicates). (**C**) Incorporation of ^14^C-labelled amino acids into the deposited ECM was measured following stimulation of human RPTEC with differing concentrations of TGFβ1. The dose-response curve shows the radioactivity count for the ECM normalised to the total cell count for the radioactive endpoint (mean ± SD of 8 replicates). Figures (**B**) and (**C**) show representative curves from at least 3 independent experiments and data from the individual repeats is summarised in Table 1.

Using RPTEC as a model system, we directly compared quantitation of ECM deposition measured by *in situ* fluorescent staining with the gold-standard radioactive incorporation endpoint. Cells were treated with varying concentrations of TGFβ1 and the induction of ECM deposition was evaluated via the two different readouts. To compensate for any effect of the stimulus on cell proliferation, signals arising from the endpoints were normalised to take account of cell number: the Flamingo fluorescent signal was normalised against cell viability determined using PrestoBlue, whilst the counts per minute (cpm) arising from the incorporation of ^14^C-labelled amino acids in the accumulated ECM was expressed relative to the cellular cpm. The extent of matrix deposited in response to TGFβ1 and stained *in situ* with Flamingo fluorescent stain showed a dose-dependent increase (Fig. 2A), which could be converted to numerical values of mean fluorescence intensity through high-content analysis of the images acquired (Fig. 2B). There was a similar dose-dependent relationship between the extent of matrix accumulation detected via incorporation of ^14^C-labelled amino acids and the concentration of TGFβ1 used as the stimulus (Fig. 2C).

TGFβ1 was a potent stimulus of ECM deposition by RPTEC and the EC_50_ values obtained for the Flamingo and radioactive assays were approximately 15 ng/mL and 4 ng/mL, respectively. The values obtained across a number of independent experimental repeats for each endpoint are summarised in Table 1.

**Table 1:** Summary of dose-response data obtained using TGFβ1 and TGFβ antibody in radioactive and Flamingo assays.

	TGFβ1		TGFβ antibody	
	EC_50_ (ng/mL)		EC_50_ (nM)	
	Flamingo	Radioactive	Flamingo	Radioactive
Geometric mean	15.0	3.7	42.6	19.3
Min, max (n)	12.5, 17.7 (4)	2.7, 4.5 (3)	20.8, 64.9 (6)	13.9, 28.0 (4)

### Modulation of deposited ECM can be measured by fluorescent staining

Considerable efforts are being made to discover and develop novel drugs to modulate ECM deposition for the treatment of fibrosis. We therefore investigated the applicability of the Flamingo *in situ* fluorescent staining method as a way to characterise modulators of ECM deposition, and compared it to the gold-standard radioactive amino acid incorporation method.

**Figure 3:**
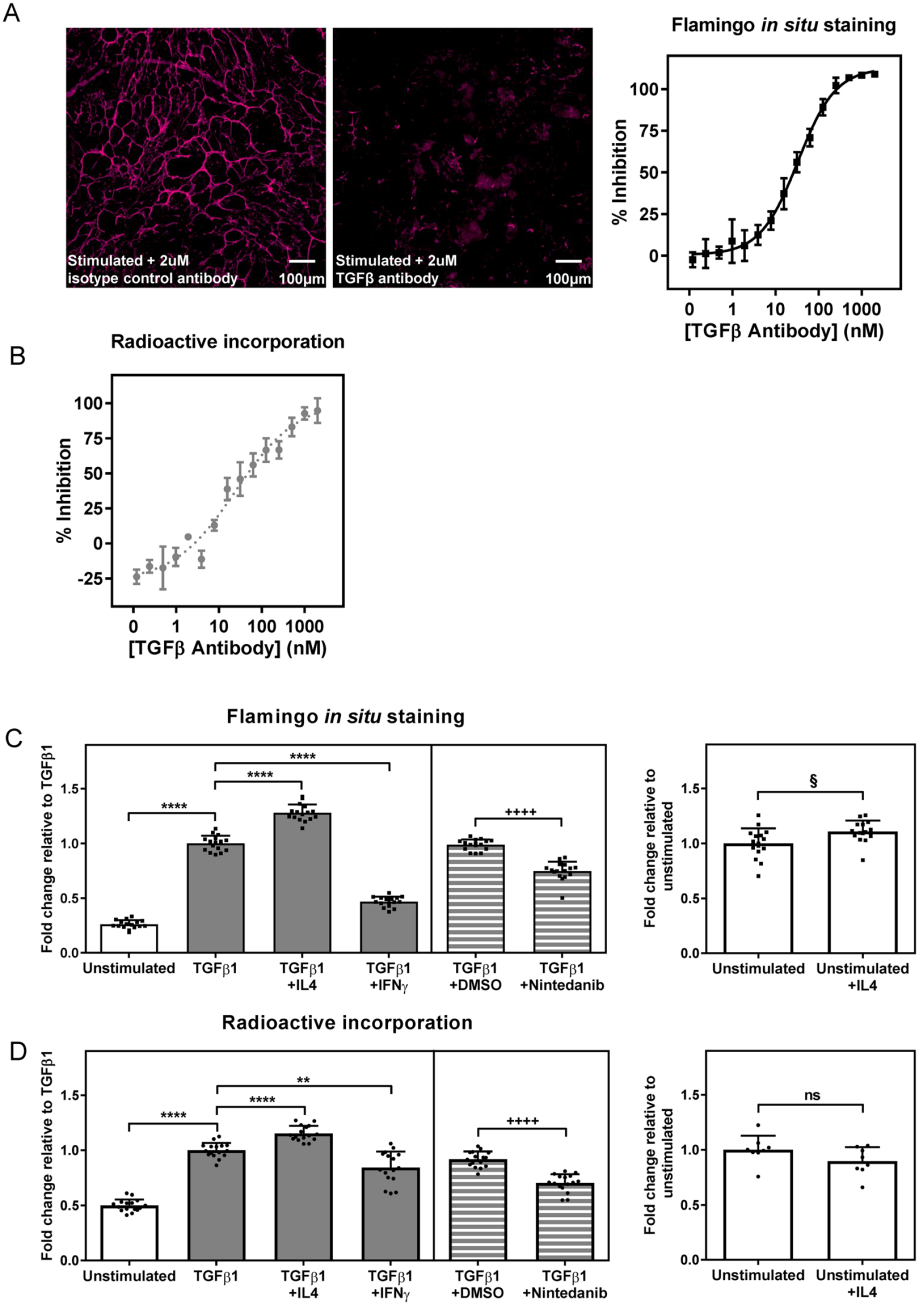
Positive and negative modulation of TGFβ1-induced ECM accumulation can be measured via *in situ* fluorescent staining with Flamingo or incorporation of ^14^C-labelled amino acids. Human RPTEC were stimulated with TGFβ1 (30 ng/ml) and the effect of different concentrations of a pan-TGFβ antibody on mature ECM accumulation determined. (**A**) Decellularised ECM was stained *in situ* using the Flamingo fluorescent stain, followed by high content image analysis. Data show percent inhibition of the mean target fluorescence intensity for ECM normalised to the PrestoBlue signal as mean ± SD of 4 replicates from a representative of 6 independent experiments. Images representative of a single field are shown. (**B**) ^14^C-labelled amino acid incorporation into deposited ECM was measured. Data show percent inhibition of the radioactivity count for the ECM normalised to the total cell count as mean ± SD of 4 replicates from a representative of 4 independent experiments. The effect of cytokines (10 ng/mL) or nintedanib (1 μM) on ECM accumulation in response to stimulation of RPTEC with 10 ng/mL TGFβ1 was determined via Flamingo *in situ* staining (**C**) or incorporation of ^14^C-labelled amino acids (**D**). For each graph, the unstimulated control is shown by the clear bar; grey bars indicate stimulation with 10 ng/mL TGFβ1 whilst striped bars indicate stimulation with 10 ng/mL TGFβ1 in the presence of 0.1% DMSO. Data show mean ± SD from a representative of four independent experiments (replicates shown as individual data points). ****P < 0.0001; **P < 0.01; one way ANOVA Vs. TGFβ1. ^++++^P < 0.0001; one way ANOVA Vs. DMSO. ^§^p < 0.05; ns, not significant; Student’s t-test Vs. unstimulated.

First, we measured ECM deposition by RPTEC stimulated with TGFβ1 in the presence of different concentrations of a pan-TGFβ neutralising antibody. The antibody dose-dependently inhibited accumulation of mature ECM in response to TGFβ1 stimulation as detected by both the Flamingo and radioactive amino acid incorporation endpoints, producing IC_50_ values of approximately 43 nM and 19 nM, respectively (Fig. 3A,B). Table 1 summarises the values obtained across a number of independent experimental repeats for each endpoint. Inhibition of TGFβ1-induced matrix deposition was specific to blockade of TGFβ1 since an isotype control antibody had no such effect.

IL4 has been identified as a pro-fibrotic mediator and elevated IL4 is associated with cardiac fibrosis^10^ whilst the Th-1 cytokine IFNγ is known to have anti-fibrotic functions^11^. We tested the effect of these cytokines, as well as the anti-fibrotic agent nintedanib, on TGFβ1-mediated ECM accumulation by RPTEC using the *in situ* fluorescence staining and radioactive amino acid incorporation endpoints, showing that each assay was capable of demonstrating positive or negative modulation of the TGFβ1-induced mature matrix accumulation (Fig. 3C,D). *In situ* staining using Flamingo showed that in the presence of TGFβ1, IL4 promoted a further increase of approximately 28% in ECM accumulation whilst in the absence of exogenous TGFβ1, the effect of IL4 on basal ECM accumulation was more modest (10% increase).

Together, these data demonstrate that fluorescent *in situ* staining of decellularised mature ECM can be used to investigate how naturally occurring mediators affect matrix accumulation, or for the screening of candidate therapeutic molecules.

### Fluorescent staining reveals organisational differences in deposited ECM

Whilst the levels of ECM accumulation directly affect scar tissue formation, one of the aspects we wanted to determine was whether this model could also be used to evaluate structural changes within the matrix. Such changes are highly relevant to the pathology of fibrosis since they can affect membrane function and local cell biology. To address this, we used the BMP antagonist Gremlin-1, which is an important pro-fibrotic mediator of TGFβ1-induced fibrosis in renal cells^12^.

**Figure 4:**
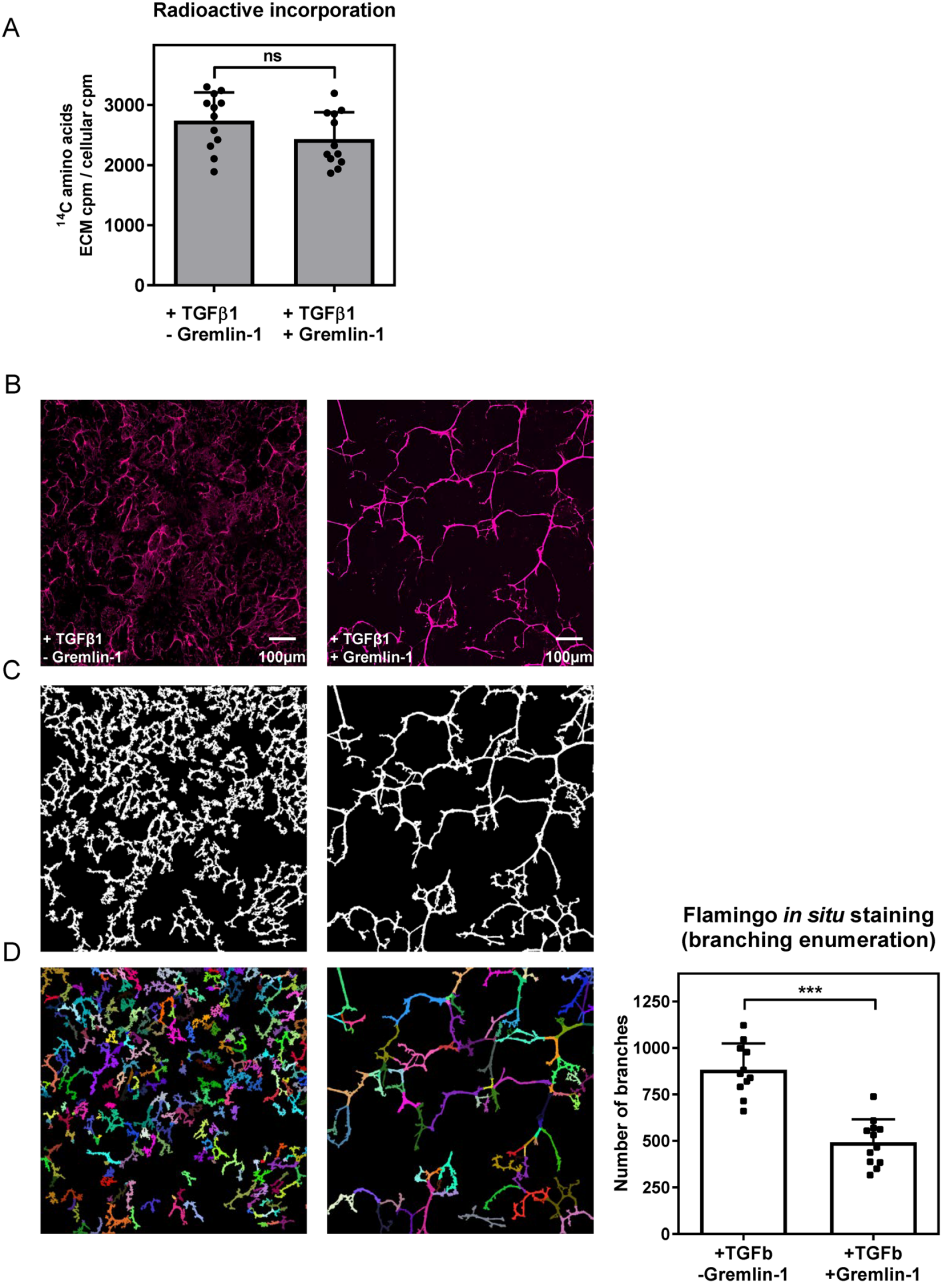
Gremlin-1 affects the nature, but not the quantity, of mature ECM accumulated by RPTEC. The effect of recombinant human Gremlin-1 on accumulation of mature ECM by RPTEC stimulated with 10 ng/mL TGFβ1. (**A**) The extent of ECM accumulation was measured via incorporation of ^14^C-labelled amino acids. Data show mean ± SD from three independent experiments (individual data points are also shown). ns, not significant; Student’s t-test. (**B**) Decellularised ECM was fluorescently stained *in situ* using Flamingo. Images show a single field. (**C**) Flamingo-labelled ECM images were segmented from the background with analysed region shown in white and branching points within the ECM were analysed using Pipeline Pilot with branches highlighted in unique colours (**D**) and the number of branches formed in the absence or presence of Gremlin-1 was plotted. Data show mean ± SD from a representative experiment (12 replicates shown as individual data points). ***P < 0.001; Student’s t-test.

The bioactivity of recombinant human Gremlin-1 was confirmed using an *Id1* reporter assay (Supplementary Fig. 5) and the effect of Gremlin-1 on ECM accumulation in each of the ECM accumulation assays was examined. In the absence of TGFβ1, Gremlin-1 had a minimal effect on matrix accumulation, and the inclusion of Gremlin-1 with TGFβ1 did not change the extent of mature ECM accumulated, as measured via incorporation of ^14^C-labelled amino acids (Fig. 4A). In contrast, *in situ* fluorescent staining revealed that Gremlin-1 promoted a noticeable alteration in the fibrillary nature of the matrix deposited in response to TGFβ1 (Fig. 4B). As compared to the ECM laid down in response to TGFβ1 – which displayed the characteristic major fibres linked by a network of smaller fibres – matrix accumulated in the presence of Gremlin-1 was more sparsely dispersed and was composed of thicker fibrils which were less extensively connected and the mesh of fine interlinking fibres was no longer observed. In order to quantitate this effect, Flamingo-labelled ECM images were segmented from the background with the analysed region shown in white (Fig. 4C) and Pipeline Pilot software used to analyse branching points within the ECM (Fig. 4D shows individual branches highlighted in unique colours). This analysis permitted enumeration of the branches present in ECM deposited by TGFβ1-stimulated RPTEC (Fig. 4D). Gremlin-1 significantly reduced the number of branches (P < 0.001) suggesting that the organisation of the branches had been rearranged, rather than the amount of ECM increased or decreased through treatment. The ability to fluorescently stain *in situ* decellularised ECM in this way allowed quantitation of the effect of Gremlin-1 on the ECM, which could not be measured using the radioactive incorporation assay since this modulator changed the nature, but not the total amount, of matrix accumulated.

## Discussion

The *in vitro* platforms available for drug discovery have advanced significantly in recent years. In the cell biology arena, the shift has been away from using highly engineered cell lines for target-based drug discovery, towards phenotypic screens using physiologically relevant animal or human cells. This approach introduces more aspects of complex physiology/pathology whilst maintaining the ability to perform high-throughput screening of novel biological or chemical entities. In an effort to increase the translation from animal models into human disease, early screens often employ both animal and human cells, and primary cells are increasingly used to model key aspects of disease biology. Hence, methods which offer cross-species endpoints without the need for additional optimisation will facilitate rapid parallel screening and increase translational confidence.

There is currently great interest within the drug discovery arena around fibrosis. This is a common pathological feature of many diseases which occurs in nearly all organs, including lung, heart, skin, muscle, liver, kidney and pancreas, and is associated with substantial morbidity and mortality^3^. Organ failure resulting from fibrosis has been estimated to account for at least one third of deaths worldwide^13^. Although progress has been made in the treatment of fibrotic diseases, most notably with the approval of the anti-fibrotic drugs pirfenidone and nintedanib for the treatment of IPF in 2014^14^, there is a great need for additional therapies outside this one indication. Therefore, the basic biology underlying the regulation of ECM deposition is an area of major research, and increased understanding of the underlying mechanisms will uncover novel points of intervention for the treatment of fibrotic and other connective tissue diseases.

The ECM is a highly complex and highly dynamic system of glycoproteins and proteoglycans; it is processed by proteolytic enzymes and provides a structural framework for the organisation of tissues and organs^1,2^. ECM homeostasis is maintained in healthy tissue through continuous remodelling, in which protein turnover is kept under tight control^15^. In contrast to the relatively stable composition of ECM in healthy adult tissue, fibrotic disease is marked by changes in the ECM balance and in particular, excessive production and accumulation of fibrillary collagens. Within this context, TGFβ has been identified as a pivotal agent in fibrosis^7–9^. Changes in ECM in fibrosis reflect alteration in the expression of matrix components, as well as dysregulation of the balance between proteolytic enzymes and their inhibitors. Expression of extracellular matrix proteins and their processing enzymes is subject to epigenetic regulation^16^ and post-transcriptional regulation of matrix components by miRNAs has been intimately linked with TGFβ1 signalling^17,18^. Fibrotic ECM can promote a feedback loop in which the matrix regulates miRNA expression in cells contacting the ECM, and those miRNAs modulate expression of key ECM components^18^, thus indicating a fundamental interplay between aberrant ECM and the cells it contacts. Although the collagens and glycoproteins such as fibronectin are the major protein components of mature ECM^2^, the ECM proteome is diverse: mass spectrometry-based proteomics revealed the presence of more than 140 proteins in the basement membrane of healthy renal glomerulus^19^. Matrix constituents and organisation also vary with tissue type, which reflects the differing mechanical and chemical properties required by that particular environment (reviewed by refs^2,5,15,20–23^). Methods are therefore required which can quantify the dysregulation of mature matrix accumulation in fibrotic diseases.

Accumulation of mature ECM *in vitro* can be measured via incorporation of radioactive amino acids into the matrix. However, this method is reliant on the use of costly unstable isotopes and although we have shown that it may be effectively miniaturised into 96- or 384-well formats, it is not well-suited to robotic automation. Alternatively, immunofluorescence techniques can be used to monitor ECM components, but this approach requires significant optimisation and the number of proteins which can be monitored is constrained by the availability of suitable antibodies as well as the limited multiplexing capabilities of high-content microscopes. The measurement of a small subset of proteins in the matrix may overlook its total protein composition and therefore could give rise to false negatives during screening. We showed that fixed decellularised ECM deposited by a variety of human and animal cell types could be stained *in situ* using the Flamingo fluorescent reagent. The fibrillar nature of the ECM stained in this manner is reminiscent of the ECM deposited *in vitro* by podocytes or glomerular endothelial cells^5^, or by dermal fibroblasts co-cultured with MDA231 breast cancer cells^6^, and *ex vivo* denuded amniotic membrane^24^. The data we generated via *in situ* staining of accumulated ECM with Flamingo compared well with the more traditional radioactive endpoint. This novel approach enables the quantitation of accumulated mature ECM by cell types from different organs as well as from different species, facilitating measurement of the effect of both biological and chemical modulators on the ECM in a high-throughput manner. Whilst it is not yet possible to mirror the complete process of fibrosis *in vitro*, we believe our *in situ* staining approach offers an improvement over current methodologies. We used this novel method within our laboratories for library screening, and achieved > 60% concordance with hits from an *in vivo* model of organ fibrosis, thus demonstrating the translational relevance of this *in vitro* endpoint.

The orientation and stiffness of ECM fibres influences cell migration and adhesion^5,22^, and mechanical testing demonstrates that the ECM of fibrotic tissue is stiffer than the corresponding healthy tissue, hence revealing increased matrix stiffness as a hallmark of organ fibrosis^25^. Pathological stiffening of ECM causes aberrant cellular mechanosensing which in turn promotes increased invasion of exogenous cells including immune cells and myofibroblasts, along with enhanced motility of resident cells^25^. Increased matrix stiffness reflects an elevation in type I collagen content as well more extensive cross-linking through the actions of enzymes such as lysyl oxidase and transglutaminase^2^. Thus, the physical and organisational properties of the ECM are highly relevant to its effects on the cells it contacts. In addition to demonstrating quantitative differences in matrix deposition, our approach of fluorescent *in situ* staining allowed us to detect differences in the organisation of the deposited ECM. We observed a pronounced change in the nature, but not the total amount, of mature matrix accumulated by RPTEC stimulated by TGFβ1 when cultured in the presence of the cysteine knot protein Gremlin-1. A similar change in ECM organisation following treatment with Gremlin-1 has been previously reported^12^ and we further investigated this effect through *in situ* fluorescent staining of decellularised ECM. Instead of the fine mesh of ECM which was laid down in response to TGFβ1 stimulation, the fibrils of the Gremlin-1 modulated matrix were fewer in number and showed decreased branching which could be quantified by analysis of the images. The ECM fibrils deposited in the presence of Gremlin-1 also appeared thicker; whilst we did not quantitate this parameter, it is an additional read-out offered by this *in situ* staining method. The type of changes observed in mature ECM accumulated in the presence of Gremlin-1 may contribute to the pro-fibrotic actions of Gremlin-1^12^ and could not be detected using the radioactive amino acid incorporation assay.

This advance in the detection of deposited ECM through fluorescent *in situ* staining may be compared to the progress in proteomics which occurred when such stains superseded the complex method of silver staining to detect proteins separated through gel electrophoresis. The Flamingo fluorescent gel stain that we utilised is a coumarin-based cyanine dye which gives a low background signal and fluoresces strongly upon binding to fixed protein^26^. Furthermore, it is highly sensitive; being able to detect 30 pg of denatured protein, and also allows linear quantitation over more than three log orders of magnitude^27–29^. In a comparison with another widely used proteomics gel stain, SYPRO Ruby, the Flamingo stain was found to detect a greater number of proteins in tissue lysates separated by 2D gel electrophoresis^30^, indicating superior sensitivity. The existence of protein-to-protein variation is a known property of proteomics gel stains^30^ and although reduced staining intensity of the highly acidic protein pepsin is reported with most fluorescent gel stains, including SYPRO Ruby, Deep Purple and Flamingo, in a comparison of these reagents, Flamingo exhibited the lowest average mean staining deviation across a range of proteins of different size, isoelectric point or post-translational modification^29^. A further potential limitation with this technique lies in the algorithms used to process the images; however the application of more sophisticated analyses of the acquired images will address some of these challenges.

The novel ECM staining method we have described here is exemplified for the most part using a renal epithelial cell model of fibrosis, but importantly, it is equally applicable to other *in vitro* cell-based models in which matrix protein deposition reflects the underlying biology or pathology. In addition to the central involvement of the ECM in organ fibrosis and cancer metastasis, conditions such as osteogenesis imperfecta, epidermolysis bullosa and Ehlers-Danlos syndrome reflect defects in ECM quality whilst properly functional ECM remodelling is required for angiogenesis and wound healing^1,6,9,31^. We have generated preliminary data which supports the applicability of our novel *in situ* fluorescent staining method to *in vitro* models for two of these additional settings. Supplementary Fig. 6 shows that the ECM deposited by mouse pre-osteoblast MC-3T3 cells revealed by *in situ* fluorescent staining is fibrillary when cells are cultured in expansion medium, but more closely resembles mineralised matrix when cells are cultured under osteogenic conditions. Deposition of mature matrix by HUVEC has been shown to be regulated by oxygen tension^32^; Supplementary Fig. 7 demonstrates the ability of our methodology in this setting and reveals hypoxia as a potent stimulus of ECM deposition by HUVEC. Together, these additional data demonstrate that *in situ* fluorescent staining of deposited mature ECM is applicable to models beyond fibrosis. Hence, the approach we describe here offers a new method to reveal mechanisms of regulation and modulation of accumulated mature ECM matrix which will give insights into basic biology, as well as providing a convenient high-throughput platform for the discovery of potential therapeutic agents.

## Methods

### Cell lines and reagents

Human primary RPTEC were purchased from InnoProt (P10662) and were cultured in Renal Epithelial Cell Basal Medium (ATCC PCS-400-030) supplemented with Renal Epithelial Cell Growth Kit Components (ATCC PCS-400-040) in 100% humidity and 5% CO_2_ at 37 °C and were used until passage 6.

Human primary IPF134 cells were from ATCC (CCL-134) and were maintained in Primary Fibroblast Media (Lonza FGM-2 Bulletkit CC-3132).

Rabbit primary RPTEC and fibroblasts were isolated from the kidneys of New Zealand White rabbits. Rabbits were maintained at temperature between 17 °C to 19 °C with humidity of 40–60% on a 12-hour light/dark cycle. Animals were allowed free access to tap water and SAFE diet supplemented with hay and peas. All procedures were carried out under license according to UK guidelines (Animals (Scientific Procedures) Act 1986) and were approved by the UCB Pharma Animal Welfare and Ethical Review Body. Briefly, the animals were anaesthetised and terminated using an injection of Euthetal (Merial) into the ear vein. Cessation of brain activity (loss of reflexes) and cessation of circulation were used to confirm death. The kidneys were removed immediately washed in ice-cold PBS (Gibco) containing PenStrep and fungicide (Sigma). The cortices of the kidneys, which contained the tubules and glomeruli, were isolated and minced into 1 mm^3^ pieces and incubated with 1 mg/mL collagenase (Sigma) at 37 °C for 30 minutes. This was pulped (squashed) and the cortical pulp was pushed through successive sieves (300 µm then 180 µm) to isolate the tubules which were retained on the 180 µm sieve whilst the glomeruli were washed off into the waste. The tubular fractions were grown in Renal Epithelial Cell Basal Medium (ATCC PCS-400-030) and Primary Fibroblast Media (Lonza FGM-2 Bulletkit CC-3132) in 100% humidity and 5% CO_2_ at 37 °C to selectively isolate populations of tubular epithelial cells and fibroblasts.

All primary cells were transferred into Renal Epithelial Cell Basal Media (ATCC PCS-400-030) supplemented with Renal Epithelial Cell Growth Kit (ATCC PCS-400-040) for assays. Cells were stimulated with 10 ng/mL TGFβ1 (Peprotech) unless otherwise stated. IL-4 and IFNγ (Peprotech) were added at 10 ng/mL and Gremlin-1(R&D Systems) was added at 1 μg/mL. For TGFβ1 blockade, anti-TGFβ (R&D Systems, MAB1835) or the corresponding isotype control (R&D Systems, MAB002) were included at the indicated concentrations. The compound nintedanib (Selleck Chem) was prepared in DMSO and used at 1 μM (final concentration of DMSO was 0.1%).

Flamingo fluorescent gel stain was obtained from BioRad and SYPRO Ruby was from ThermoFisher Scientific.

### mRNA analysis

Cells (2.5 × 10^5^/well) were cultured for 48 hours in 6-well plates, in the presence or absence of 10 ng/mL TGFβ1. Medium was removed and the cell monolayer washed once with cold PBS. Total RNA was isolated using RNEasy Plus Mini kit (Qiagen), in accordance with the manufacturer’s instructions. The resultant RNA was quantitated by spectrophotometry before being reverse transcribed using SuperScript VILO cDNA synthesis kit (Life Technologies) in accordance with the manufacturer’s instructions. Quantitative real-time PCR (qRT-PCR) was performed in triplicate wells using TaqMan Gene Expression Master Mix (Life Technologies) with 1 μL cDNA/well. No RT and no template control wells were included in each experiment. The ΔΔCt method was used to normalise expression against the geometric mean of the housekeeper genes B2M, HMBS and TBP. TaqMan probes (Life Technologies) are shown in Supplementary Table S1.

### Radioactive ECM deposition assay

Cells (5 × 10^3^/well for 96-well plates, 2 × 10^3^/well for-384 well plates) were seeded in Cytostar-T plates (Perkin Elmer) and were cultured in media supplemented with 0.75 µCi/mL of ^14^Carbon L-Amino Acid mixture (Hartmann Analytic) for 7 days. Cells were removed by lysis with 0.25 M ammonium hydroxide in 25 mM Tris pH7.4 (as described^33^); the matrix washed with PBS, and radioactivity measured using a TriLux 1450 Microbeta Scintillation Counter (Perkin Elmer).

### Measurement of cell growth

Cell growth was assessed using PrestoBlue Cell Viability reagent (Thermo Fischer Scientific) following the instructions of the manufacturer.

### Fluorescent staining of ECM

Cells (5 × 10^3^/well for 96-well plates, 2.5 × 10^3^/well for 384-well plates) were plated into dark walled imaging plates (BD Biosciences or Greiner). Following stimulation and 7 days incubation at 37 °C 5% CO_2_, cells were washed in PBS and lysed with 0.25 M ammonium hydroxide in 25 mM Tris for 15 min at 37 °C. The matrix was washed 3 times in PBS, fixed using 100% methanol (unless otherwise stated) for 30 min at -20 °C and washed 3 times in PBS before being stained overnight using 1X Flamingo fluorescent stain.

### Image acquisition and analysis

Images were acquired using a Cellomics Arrayscan HC reader (Life Technologies) using a 10x Objective, ORCA-ER or X1 camera with 2 × 2 binning (1104 × 1104 pixels/field). Flamingo-stained ECM was detected using 485 nm excitation and 607 nm emission filter sets. For analysis of extracellular matrix fibrils we used the ‘Cell Health Profiling’ algorithm and used the entire image as the object. Staining above a fixed threshold was analysed and total intensity of the target was measured.

#### Matrix branching analysis

Image analysis of ECM stained with Flamingo was conducted using Pipeline Pilot 9.2 (Biovia, US). Images from the four fields within each well (384-well plate) were tiled together before an adaptive segmentation threshold was applied to each individual pixel. The segmented regions were skeletonized using a fast marching distance transform to form markers regions which were subsequently grown into the surrounding area on the original image using a flood fill from markers algorithm. ECM branches which fulfilled the inclusion criteria of having mean pixel intensity of +150 or -50 from the marker region were individually labelled and the number and average area of the branches within a well were determined.

### Data analysis and availability

Statistical analysis was performed using GraphPad Prism 6.05. Comparisons of data were performed using an unpaired Student’s t-test or one-way ANOVA with Dunnett’s post-hoc test at each time point; P < 0.05 was considered statistically significant. All graphical data are presented as mean ± SD.

The datasets generated during and/or analysed during the current study are available from the corresponding author on reasonable request.

## Electronic supplementary material

Supplementary Information
