# Publisher Correction: Quantitative and organisational changes in mature extracellular matrix revealed through high-content imaging of total protein fluorescently stained *in situ*

**DOI:** 10.1038/s41598-017-16513-z

**Published:** 2017-12-21

**Authors:** Gill Holdsworth, Hélène Bon, Marianne Bergin, Omar Qureshi, Ross Paveley, John Atkinson, Linghong Huang, Roohi Tewari, Breda Twomey, Timothy Johnson

**Affiliations:** UCB Pharma, Slough, UK

Correction to: *Scientific Reports* 10.1038/s41598-017-10298-x, published online 30 August 2017

The original PDF version of this Article contained an error where Figure 3 was truncated. This error has now been corrected in the PDF version of the Article; the HTML version was correct from the time of publication.

