# Supplementary material for: Quantitative and organisational changes in mature extracellular matrix revealed through high-content imaging of total protein fluorescently stained in situ
Source: Sci Rep. 2017 Aug 30;7:9963. doi: 10.1038/s41598-017-10298-x (PMC5577101; doi:10.1038/s41598-017-10298-x)
Supplement: Supplementary file 1 — Supplementary Information [file 41598_2017_10298_MOESM1_ESM.pdf]

Quantitative and organisational changes in mature extracellular matrix revealed through high-content imaging of total protein fluorescently stained *in situ*.

Gill Holdsworth*[§], Hélène Bon[§], Marianne Bergin, Omar Qureshi, Ross Paveley, John Atkinson, Linghong Huang, Roohi Tewari, Breda Twomey, Timothy Johnson

[§] These authors contributed equally to this work

* Corresponding author

Gill.Holdsworth@ucb.com

Author affiliations: UCB Pharma, Slough, U.K.

Gene symbol	Gene name	TaqMan probe
Col1a1	Collagen, type I, alpha 1	Hs00164004_m1
Col3a1	Collagen, type III, alpha 1	Hs00943809_m1
Col4a1	Collagen, type IV, alpha 1	Hs00266237_m1
Fn1	Fibronectin 1	Hs01549976_m1
B2M	Beta-2-microglobulin	Hs00187842_m1
HMBS	Hydroxymethylbilane synthase	Hs00609296_g1
TBP	TATA box binding protein	Hs00427620_m1

Supplementary Table S1. TaqMan probes used for qRT-PCR experiments

A

	Well surface area	Cells/well	Radioactivity/well	Well assay volume
6-well plate	962mm^2	200 x 10^3	5µCi	3mL
24-well plate	200mm^2	40 x 10^3	1µCi	625µL
96-well plate	47mm^2	5 x 10^3	0.15µCi	200µL
384-well plate	13mm^2	2 x 10^3	0.0375µCi	50µL

B

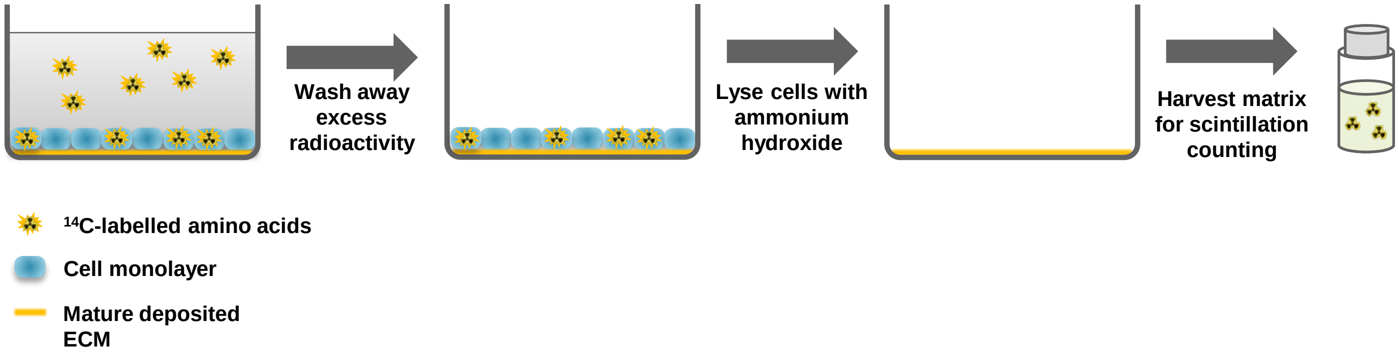

C

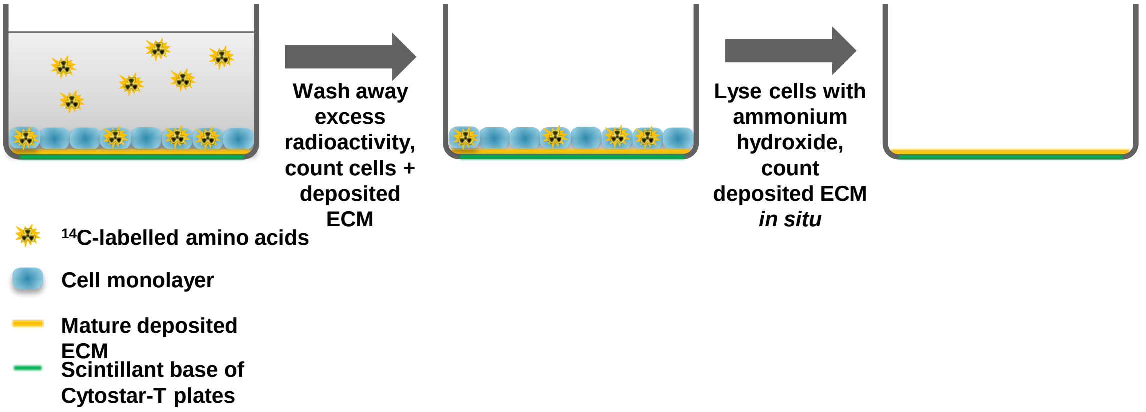

Supplementary Figure S2. Miniaturisation of the radioactive ECM accumulation assay
The method to determine incorporation of radiolabelled amino acids into accumulated mature ECM was miniaturised successively using the culture areas and cell densities shown in **A**. The first phase of this assay optimisation used the traditional methodology: following culture of cells in media containing ^{14}C-labelled amino acids, excess radioactivity was removed by washing and cells lysed as described in materials and methods; the cell lysate was retained for protein measurement by BCA assay. Following extensive washing with PBS to remove cell debris, ECM was harvested using SDS or enzymatic methods and the radioactive decay of the solubilised ECM measured by liquid scintillation counting as per the scheme shown in **B**. In a further development, which removed the need for matrix harvest altogether, the assay was transferred to CytoStar-T scintillating microplates which use the principles of scintillation proximity assay technology, hence allowing the deposited matrix to be measured *in situ*, shown in **C**. This high-throughput approach was successfully applied in 96- and 384-well plate formats.

A

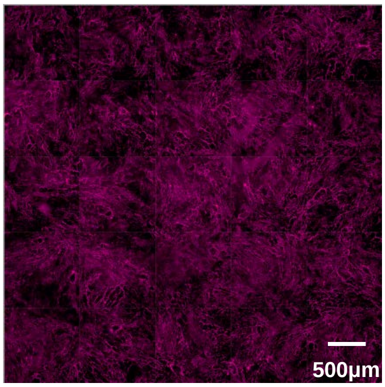

B

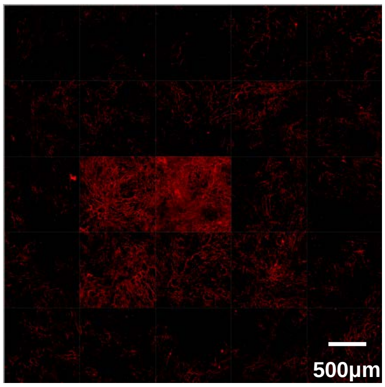

C

25	24	23	22	21
10	9	8	7	20
11	2	1	6	19
12	3	4	5	18
13	14	15	16	17

Supplementary Figure S3. *In situ* staining of decellularised ECM using SYPRO Ruby is subject to photobleaching

Photobleaching of decellularised ECM stained *in situ* was observed with SYPRO Ruby but not Flamingo. RPTEC in a 96 well plate were stimulated for 7 days with 10ng/mL TGFβ1. Matrix was decellularised as described in Materials and Methods and following fixation with 50% methanol/7% acetic acid for 2 hours, ECM stained *in situ* overnight using Flamingo (**A**) or SYPRO Ruby (**B**) was visualised using fluorescence microscopy. The entire well was excited and all 25 fields captured in a counter-clockwise spiral, starting from the centre of the well (field 1), as illustrated by the schematic (**C**).

There was marked bleaching of the outer fields in the wells stained with SYPRO Ruby but this did not occur in wells stained with Flamingo. The images in **A** and **B** show the entire well as a composite of the individual fields acquired from that well.

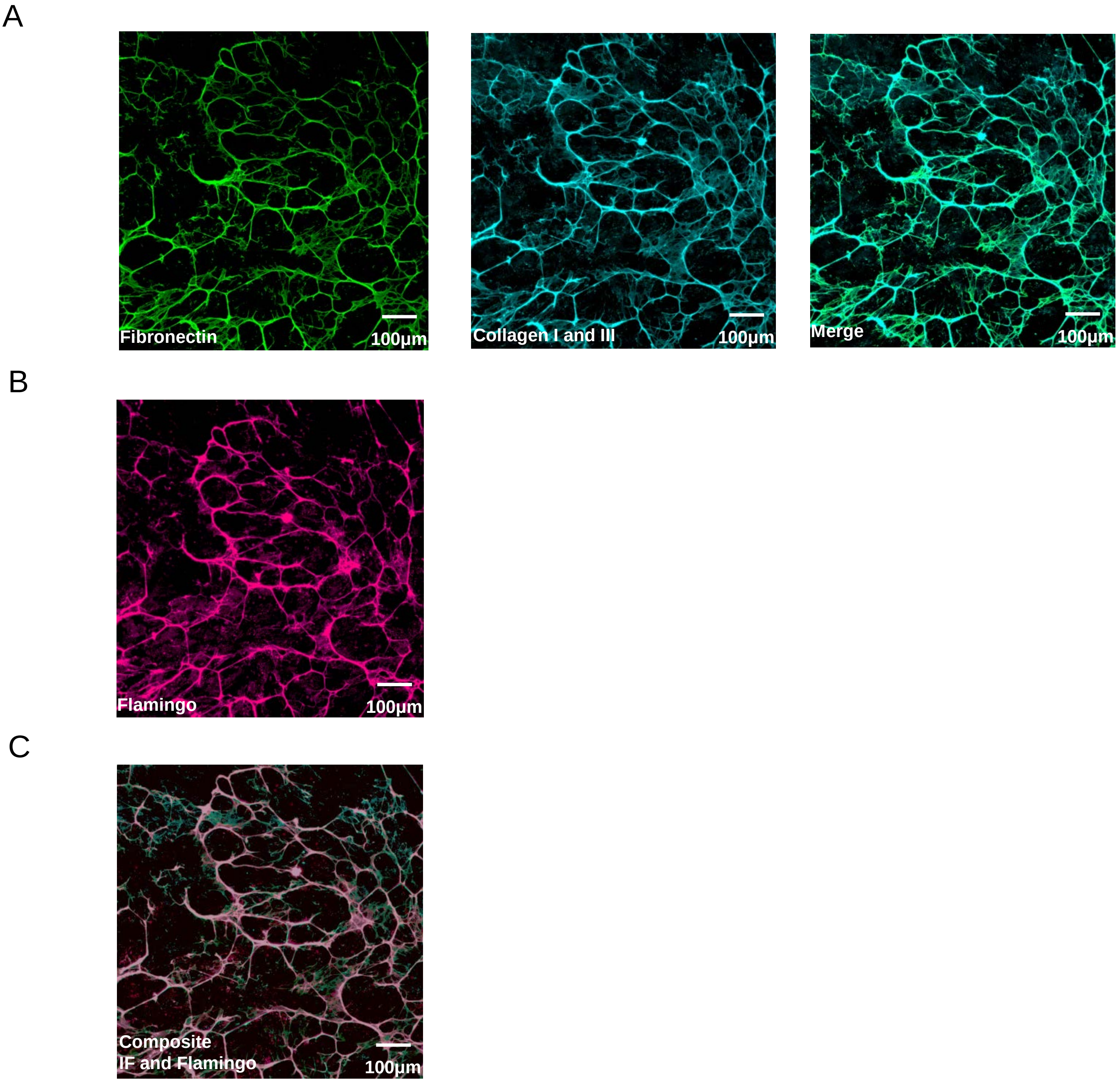

Supplementary Figure S4. ***In situ*** **fluorescent staining of decellularised ECM using Flamingo co-localises with matrix detected by collagen type I/III and fibronectin immunofluorescent staining**
(**A**) RPTEC were cultured in the presence of 10ng/mL TGFβ1 for 7 days. Fixed decellularised matrix was stained *in situ* with anti-fibronectin (EBioscience, clone FN-3) and anti-collagen I and III (Merck Millipore, AB745 and AB747). Anti-fibronectin was directly conjugated (Alexa Fluor488); anti-collagen type I and III were detected using Alexa Fluor647-conjugated anti-rabbit secondary antibody (Life Technologies). Images were acquired using a Cellomics Arrayscan HC reader using excitation/emission filter sets of 485nm/525nm and 650nm/684nm for fibronectin or collagen I/III, respectively. The panel on the right shows a merge of fibronectin (green) with collagen type I/III (blue). (**B**) Post IF, matrix was restained using Flamingo; the image shows the same field as **A**. (**C**) Composite of IF merge and Flamingo images.

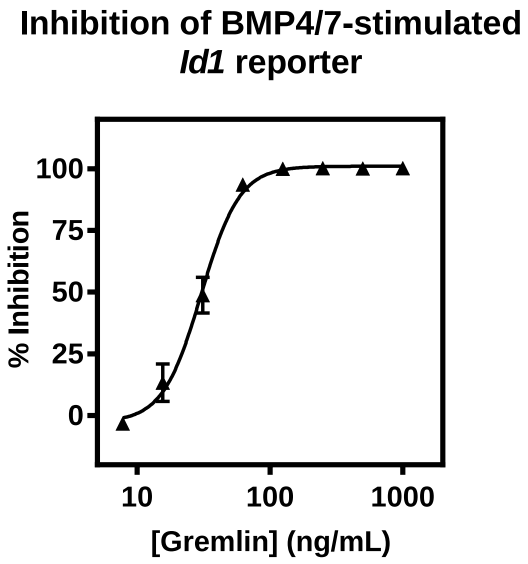

Supplementary Figure S5. Recombinant human Gremlin-1 dose-dependently inhibits the BMP4/7 response in HEK-*Id1* reporter assay

HEK293 cells stably transfected with an *Id1*-Luciferase reporter in the pGL4.26 vector (Promega) were maintained in DMEM + 10% FCS + 2mM L-Glutamine. For the luciferase assay, 5 x 10^4 cells/well were seeded into white poly-D-lysine coated 96 well plates (BD Biosciences) and incubated for 4 hours at 37°C to adhere. BMP4/7 heterodimer (R&D Systems) was reconstituted in 4mM HCl before being diluted into medium and was used at a final concentration of 100ng/mL. Gremlin-1 (R&D Systems) was added at the concentrations indicated. Plates were incubated for 24hr at 37°C before the luciferase signal was detected using SteadyGlo (Promega). Data have been normalised to show percentage inhibition of the maximal signal determined in the absence of Gremlin-1 and are plotted as mean ± SD for duplicate determinations.

A

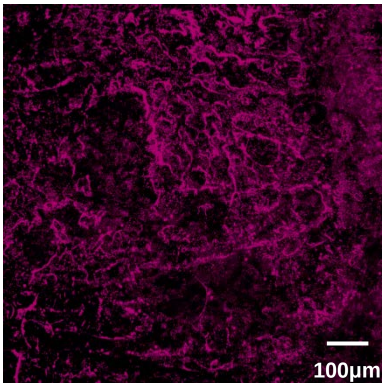

B

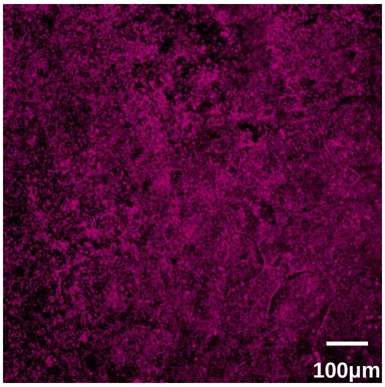

Supplementary Figure S6. Mature ECM accumulated by osteoblasts can be detected via *in situ* fluorescent staining using Flamingo

MC-3T3 mouse pre-osteoblasts were cultured in expansion medium (alpha MEM containing 2mM L-Glutamine with 10% non-heat inactivated FBS) (**A**), or osteogenic differentiation medium (expansion medium supplemented with 50µg/mL ascorbate-2-phosphate and 5mM β-glycerophosphate) (**B**), for 6 days and the ECM accumulated was stained *in situ* using Flamingo. Mature ECM accumulated in both culture conditions but the matrix observed using cells cultured in expansion medium was fibrillary in nature whilst that obtained from cells grown in osteogenic medium more closely resembled bone matrix.

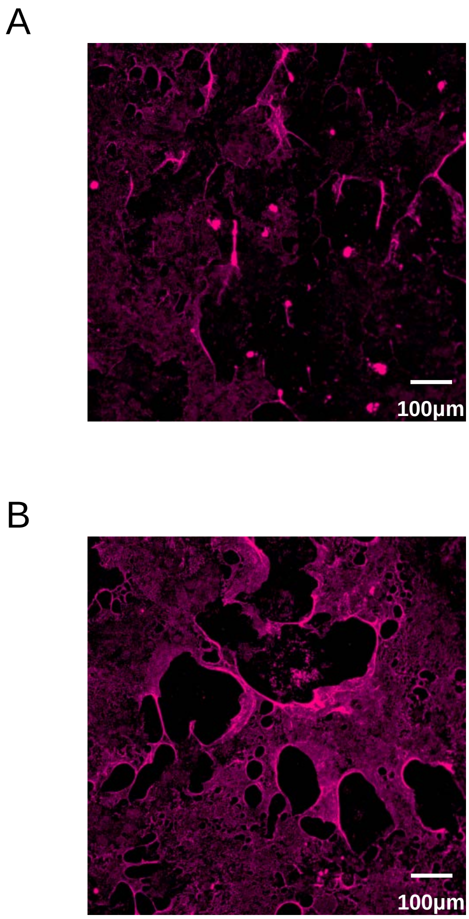

Supplementary Figure S7. Increased accumulation of mature ECM by HUVEC in response to hypoxia can be detected via *in situ* fluorescent staining using Flamingo
HUVEC were cultured in Medium 200 basal media (Gibco) supplemented with Large Vessel Endothelial Supplement, L-Glutamine and Pen/Strep (Gibco) in normoxia (**A**) or hypoxia (2.5 % O_2) (**B**), for 6 days and the ECM deposited was stained *in situ* using Flamingo. Hypoxia was a potent stimulus of ECM accumulation by HUVEC.
